# Challenges to the Emergence of Telerehabilitation in a Developing Country: A Systematic Review

**DOI:** 10.3389/fneur.2020.01007

**Published:** 2020-09-08

**Authors:** Carl Froilan D. Leochico, Adrian I. Espiritu, Sharon D. Ignacio, Jose Alvin P. Mojica

**Affiliations:** ^1^Department of Rehabilitation Medicine, College of Medicine and Philippine General Hospital, University of the Philippines Manila, Manila, Philippines; ^2^Department of Physical Medicine and Rehabilitation, St. Luke's Medical Center, Taguig, Philippines; ^3^Department of Clinical Epidemiology, College of Medicine, University of the Philippines Manila, Manila, Philippines; ^4^Department of Neurosciences, College of Medicine and Philippine General Hospital, University of the Philippines Manila, Manila, Philippines

**Keywords:** telemedicine, telerehabilitation, barriers, rehabilitation medicine, healthcare delivery, developing country

## Abstract

**Background:** Despite being known abroad as a viable alternative to face-to-face consultation and therapy, telerehabilitation has not fully emerged in developing countries like the Philippines. In the midst of the coronavirus disease 2019 (COVID-19) pandemic, wherein social distancing disrupted the in-clinic delivery of rehabilitation services, Filipinos attempted to explore telerehabilitation. However, several hindrances were observed especially during the pre-implementation phase of telerehabilitation, necessitating a review of existing local evidences.

**Objective:** We aimed to determine the challenges faced by telerehabilitation in the Philippines.

**Method:** We searched until March 2020 through PubMed, Scopus, Embase, Cochrane Library, and HeRDIN for telerehabilitation-related publications wherein Filipinos were involved as investigator or population. Because of the hypothesized low number of scientific outputs on telerehabilitation locally, we performed handsearching through gray literature and included relevant papers from different rehabilitation-related professional organizations in the Philippines. We analyzed the papers and extracted the human, organizational, and technical challenges to telerehabilitation or telehealth in general.

**Results:** We analyzed 21 published and 4 unpublished papers, which were mostly reviews (8), feasibility studies (6), or case reports/series (4). Twelve out of 25 studies engaged patients and physicians in remote teleconsultation, teletherapy, telementoring, or telemonitoring. Patients sought telemedicine or telerehabilitation for general medical conditions (in 3 studies), chronic diseases (2), mental health issues (2), orthopedic problems (2), neurologic conditions (1), communication disorders (1), and cardiac conditions (1). Outcomes in aforementioned studies mostly included telehealth acceptance, facilitators, barriers, and satisfaction. Other studies were related to telehealth governance, legalities, and ethical issues. We identified 18 human, 17 organizational, and 18 technical unique challenges related to telerehabilitation in the Philippines. The most common challenges were slow internet speed (in 10 studies), legal concerns (9), and skepticism (9).

**Conclusion:** There is paucity of data on telerehabilitation in the Philippines. Local efforts can focus on exploring or addressing the most pressing human, organizational, and technical challenges to the emergence of telerehabilitation in the country.

## Introduction

**Figure 1:**
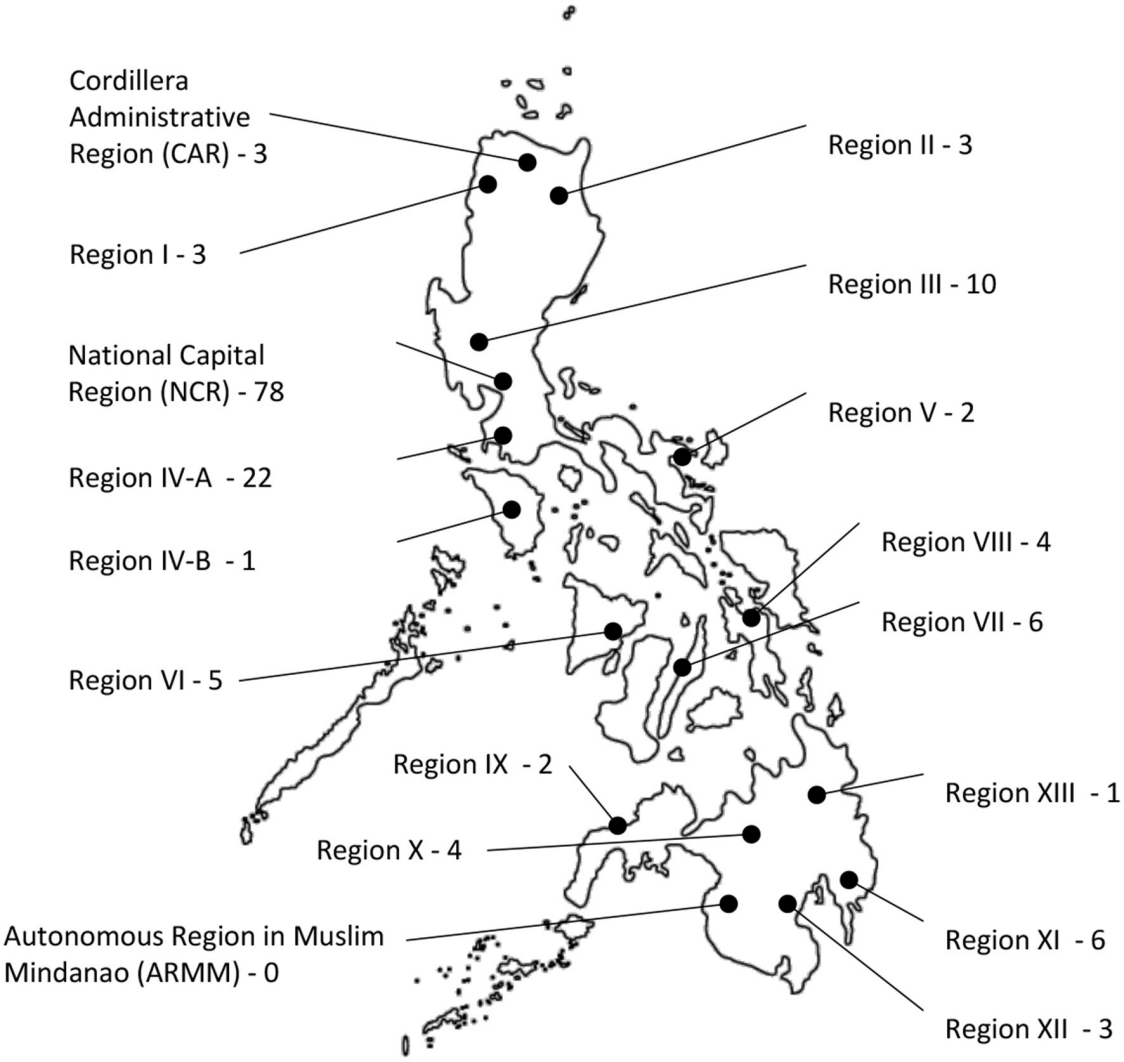
Geographical landscape of the Philippines divided into 17 administrative regions, each with a corresponding number of physiatrists in their place of primary practice.

Located in Southeast Asia, the Republic of the Philippines consists of an archipelago of 7,641 islands with a land area of more than 300,000 km^2^ (1). The country is divided into three large groups of islands, namely, Luzon, Visayas, and Mindanao, and into 17 administrative regions (Figure 1) (2, 3). The Philippine National Statistics Office estimated that the total population in the country would reach 110 million in 2020 (4). As of writing, the country has a gross national income per capita of 3,830 USD and remains to be a lower-middle-income economy according to The World Bank (5, 6). The geographical landscape, administrative organization, and growing imbalance between population and resources are among the reasons that contribute to the difficult distribution of healthcare services in the Philippines (7).

Of the estimated 1 billion persons with disabilities (PWD) worldwide, 80% come from low- and middle-income countries (8). Based on the 2017 Global Burden of Disease Study, the three leading causes of years lived with disability (YLDs) are low back pain, headache, and depression (9). The World Health Organization states that the total number of YLDs globally is “linked to health conditions for which rehabilitation is beneficial” (10). Rehabilitation is effective in improving or maintaining the functional independence and quality of life of PWD (8, 10). Despite limited reliable data documenting the need for rehabilitation in low- and middle-income countries, unique local experiences can attest to the prevailing unmet needs of the people amidst meager resources (11).

Telemedicine is the delivery of healthcare services through information and communications technology (ICT) to a different, often distant, site (12). As a telemedicine subset, telerehabilitation (telerehab) is an emerging technology that uses electronic means in remotely conducting evaluation, consultation, therapy, and monitoring to provide rehabilitation care for patients in various locations, such as home, community, nearby health facility, and workplace (11–13). Despite its growing body of literature and scope of services in other, mostly developed, countries, telerehabilitation continues to face challenges or barriers to its emergence in less-developed countries like the Philippines, albeit its practical use to address the widening gap between the supply of and demand for rehabilitation services especially during unprecedented times like the coronavirus disease 2019 (COVID-19) pandemic, wherein face-to-face access to rehabilitation services is hampered (14). In this review, we gathered evidences of previous local attempts at telerehabilitation along with other papers that could help us determine the human, organizational, and technical challenges that beset the emergence of telerehabilitation in the country.

## Methods

This review employed the Preferred Reporting Items for Systematic reviews and Meta-Analyses (PRISMA) consensus statements (15).

### Criteria for Study Selection

We considered studies based on the following inclusion criteria: (a) study investigator or population included Filipinos residing in the Philippines; and (b) intervention or topic included any telecommunication technology or process related to the remote delivery of medical or rehabilitation services (i.e., consultation, therapy, mentoring, and monitoring). Studies on telemedicine that focused on other specializations, such as dermatology, internal medicine, ophthalmology, pathology, or radiotherapy, were excluded. There was no restriction to the study design and year of publication or completion. Papers written in either English or Filipino were included, and those whose full text could not be accessed were not excluded to increase yield.

### Search Methods and Data Analysis

We searched the following electronic healthcare databases until March 2020 for relevant studies: MEDLINE by PubMed, Embase, Scopus, Cochrane Library, and Health Research and Development Information Network (HeRDIN), which is the Philippines' national repository of local studies. Both Medical Subject Headings (MeSH) and free search terms were used as follows: (“Telemedicine”[Mesh] OR “Telerehabilitation”[Mesh] OR “Remote Consultation”[Mesh] OR “Telenursing”[Mesh] OR telehealth OR telemedicine OR telerehabilitation OR telerehab OR teleneurorehabilitation OR teleconsultation OR teletherapy OR telepractice OR telepsychology OR telenursing) AND (“Philippines”[Mesh] OR Philippine^*^).

Due to hypothesized limited number of relevant publications from the Philippines, handsearching was done through the gray literature of different local rehabilitation professional organizations, namely, the Philippine Academy of Rehabilitation Medicine (PARM), the Philippine Physical Therapy Association (PPTA), the Philippine Academy of Occupational Therapists, Inc. (PAOT), the Philippine Association of Speech Pathologists (PASP), the Psychological Association of the Philippines (PAP), the Association of Filipino Prosthetists and Orthotists (AFPO), and the Philippine Nurses Association, Inc. (PNA). We contacted members or representatives from aforementioned organizations through text message, phone call, or email to request for relevant information.

We screened the titles and abstracts identified from the search. Relevant articles were obtained in full text (if available) and considered eligible if we could derive the following data for analysis: lead author, date of publication or completion, research design, population/target audience/problem identified, telemedicine or telerehabilitation method/concept, outcomes, and challenges to telemedicine or telerehabilitation cited in the results or discussion part. The challenges or barriers were identified, grouped together when applicable, and categorized according to unique human, organizational, and technical factors, based on consensus among study authors.

## Results

### Characteristics of Included Studies

**Figure 2:**
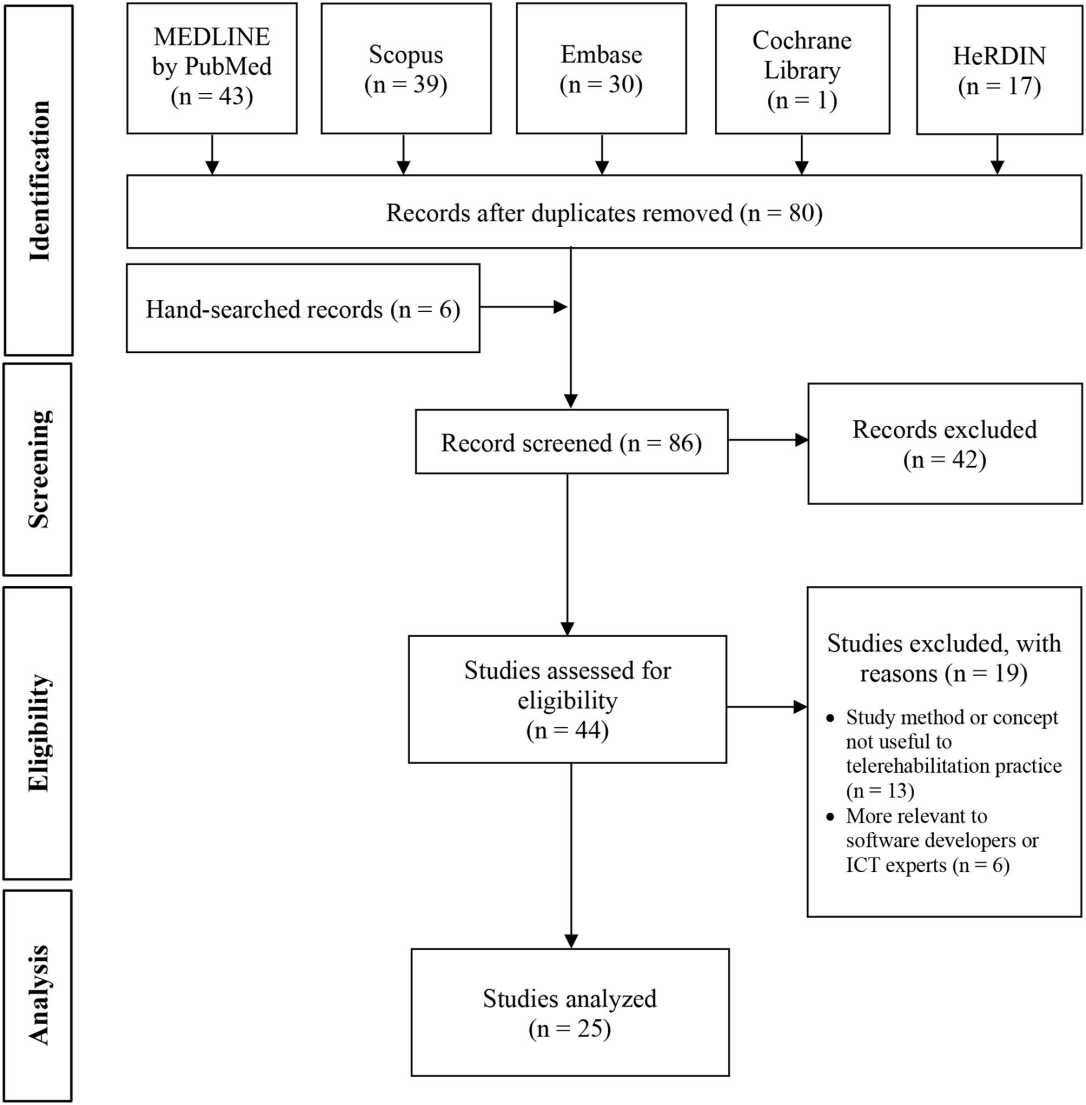
Flow diagram of study inclusion. HeRDIN, Health Research and Development Information Network; ICT, Information and communications technology.

A total of 130 documents were identified from electronic databases and 6 from handsearching (Figure 2). Fifty duplicates were discarded. Out of 86 records screened, 42 were excluded and the rest were assessed for eligibility. Nineteen articles were further excluded because of lack of relevant information. Twenty-five studies were finally analyzed.

Table 1 presents the study design, population, intervention, comparator (if any), outcomes, and challenges related to telerehabilitation of the 25 included studies (21 published and 4 unpublished). The earliest publication was in 2008, while the latest completed study was in early 2020. Studies in *Health Technology and Informatics* and *Acta Medica Philippina* were the most common journals (four studies each). There were eight review papers, six feasibility studies, and four case reports/series among other research designs, which were largely observational. Twelve out of 25 studies engaged patients from remote areas to access healthcare services or information in teleconsultation, teletherapy, telementoring, or telemonitoring. Three studies involved patients with general medical conditions (30, 33, 37), while other studies involved patients with chronic diseases (18, 29), mental health issues (17, 26), orthopedic problems (20, 28), neurologic conditions (16), communication disorders (19), and cardiac disease (36).

**Table 1:** Studies relevant to telerehabilitation with Filipinos as study lead author, co-author, or population.

**Lead author, year |** **Study design |** **Reference**	**Population/Target audience/Problem**	**Method**	**Outcomes**	**Human (H), organizational (O), and technical (T)** **challenges to telemedicine/telerehabilitation** **addressed/discussed in the study**
Leochico^a^ 2020 | Case report | (16)	2 adults with paraplegia secondary to spinal cord injury were given wheelchairs for free by a charitable institution, but were unable to comply with face-to-face wheelchair follow-up	Consultation and functional retraining through synchronous and asynchronous telerehabilitation using social media application (i.e., Viber™)	Wheelchair assessment using the World Health Organization's wheelchair follow-up form (translated into Filipino) was done through videocall. Wheelchair modifications and exercise recommendations were given. The patients were satisfied with the follow-up via telerehabilitation, obviating the need for immediate face-to-face follow-up	H: Patients' skepticism and misconceptions about telerehabilitation; lack of e-health literacy; resistance to change O: No available secure platform dedicated to telerehab; lack of local telerehab guidelines T: Slow internet
Clough^a^ 2019 | Survey | (17)	524 adults from Australia, Iran, Philippines, and South Africa with prior telemedicine experience	Online survey on potential utilization of e-mental health services	Most participants were willing to access e-health programs	H: Lack of knowledge about e-mental health services and how to access them; lack of smartphone, computer, or internet access O: Lack of methods to secure personal information; lack of freedom to use e-mental health services
Hernandez^a^ 2019 | Feasibility | (18)	Patients with chronic diseases	“Nurse Chatbot” with artificial intelligence	Chatbot can improve patient access to healthcare information	H: Lack of technology acceptance O: Lack of safeguards against privacy breach, misuse, non-transparency, abuse, human rights violation; lack of clear protocols on data encryption, cybersecurity, and informed consent T: Poor natural language processing and automated response of chatbots; conversational ambiguities; lack of empathy in e-health transactions
Ponciano-Villafania^a^ 2018 | Case report | (19)	2 elderly patients with communication disorders referred by rural-based rehabilitation specialist	Patients underwent 4 telerehabilitation sessions with a remotely located speech pathologist	Internet was slower at 7 megabits per second (Mbps) than the ideal speed (10 Mbps). Video and instant messaging using MacBook™ laptop were feasible. Participants expressed benefits from telerehabilitation	H: Lack of technical knowledge among rehabilitation providers O: Lack of telerehabilitation guidelines for full-scale implementation; lack of updated community-based rehabilitation policies and trainings T: Expensive equipment; slow internet
Leochico^a^ 2017 | Case report | (20)	Rural-based elderly post-knee arthroplasty could not access face-to-face rehabilitation services	Medical interns in the rural area referred the patient to urban-based telerehabilitation providers through Skype™	Stakeholders (i.e., patient, caregiver, students, community health workers, & telerehabilitation providers) met their needs and expectations	H: Inadequate knowledge and skills in telepresenting and telementoring O: Time-consuming in terms of setup, logistics, consultation, teaching, and technical troubleshooting; lack of professional technical support in the community; lack of exercise equipment T: Fluctuating internet; unclear video projection; unsecure videoconferencing telemedicine application
Mandirola-Brieux^b^ 2017 | Survey | (21)	Medical informatics experts	Participants were asked about their e-health perceptions on: - breaking the culture of paper - use of local language - cultural idiosyncrasies	Cultural barriers were found to be among the most important barriers in implementing e-health	H: Most doctors prefer physical records; paper culture O: Difficult e-health implementation T: Electronic records are difficult to use and sustain
Ho^c^ 2016 | Review | (22)	Policymakers, stakeholders	“Resilient health system framework” was proposed as guide to scale-up digital health and universal healthcare	The framework was built on three interlocked platforms: - leadership, policy, and governance; - health resource capacity; - information and communications infrastructure (infostructure)	H: Lack of patient engagement due to complex medical advice, poor telecommunication skills, and paternalistic medicine O: Lack of standards to ensure e-health interoperability across organizations and countries; lack of digital health policies and resources T: E-health applications lack contextualization and interoperability across practice settings and devices
Fernandez-Marcelo^c^ 2016 | Qualitative research | (23)	Policymakers, researchers, educators, healthcare providers, telehealth enthusiasts	Public fora with Department of Health, local government units, non-government organizations, academe, medical professional organizations, private sectors	Recommendations on the National Telehealth Service Program Administrative Order were given in terms of: - governance; - capacity-building; - financing; - regulation; - ethics; - data privacy	O: Lack of engagement among patient groups, clinical experts, private sectors, and local governments; lack of measures to ensure privacy and information security; unclear stakeholders' accountability; lack of providers' training, accreditation, and regulation; unclear government-subsidized financing options; lack of national policy framework; poor national ICT infrastructure
Mendoza^a^ 2016 | Review | Mendoza et al., unpublished	Problem: There is limited information about the use of telerehabilitation in developing countries	Publications on telerehabilitation across different allied health disciplines from developing countries were searched.	Publications came from Brazil, China, South Korea, South Africa, Taiwan, Hong Kong, Iran, Israel, Nigeria, Colombia, Chile, Guatemala, Nicaragua, Thailand, and Pakistan. Most studies were from higher-middle-income countries and focused on telerehabilitation interventions and assessments	H: Limited knowledge and mixed attitudes and satisfaction toward telerehab among physical therapists, occupational therapists, and speech-language pathologists; lack of acceptance; lack of personal communication/rapport; lack of digital literacy O: Lack of studies from developing countries; lack of validated data collection tools; lack of government support, continuing training, and resources; lack of guidelines to determine appropriate populations suitable for telerehab; environmental constraints T: Inability to provide manual therapy/assistance through this technology; lack of interoperability across different software applications; limited internet coverage; equipment failure
Patdu^c^ 2016 | Qualitative research | (24)	Policymakers, researchers, educators, healthcare providers, telehealth enthusiasts	Roundtable discussions with Department of Health, information technologists, lawyers	Having no law regulating telehealth in the Philippines, the following were discussed: - practice of telemedicine; - liability issues; - data privacy	O: Lack of laws governing telemedicine practice in the country; lack of security measures for sensitive information
Umali^c^ 2016 | Mixed methods | (25)	Policymakers, researchers, healthcare providers, telehealth enthusiasts	Review of policies and guidelines pertinent to e-health ethics in the Philippines; focus group discussion and key informant interviews	Gaps and lapses related to ethics were prevalent. There was a need to emphasize ethics in the development and implementation of e-health policies	O: Lack of guidelines to ensure ethical telehealth practice in the Philippines; unclear roles and liabilities
Ramos^c^ 2019 | Validation | (26)	College students	*Psychologist in a Pocket* (PiaP): mental m-health application	Significant positive correlations were found between PiaP and psychological tests. PiaP's approach to depression screening was comparable with gold standard (Beck's Depression Inventory)	H: High dropout rate in remote e-health trials; poor adherence to mental healthcare applications T: Lack of perfect correlation between the software application and face-to-face psychological tests; inability of the app to detect behavioral signs of depression, which could not be expressed through text
Laron^b^ 2015 | Review | Laron et al., unpublished	Physical therapists, occupational therapists, speech-language pathologists	Literature review on telerehabilitation knowledge, attitudes, and perceptions among therapists	Most studies utilized customized, non-validated questionnaires. Attitudes and perceptions were mostly positive. One study showed low knowledge	H: Incomplete acceptance; apprehensions related to virtual environment, rapport, accuracy, effectiveness, efficiency, suitability to healthcare needs and resources, safety O: Lack of government support, continuing training, and resources T: Inability of the technology to provide manual assistance during therapy; lack of flexibility across available telerehab software applications; limited internet coverage
Marcelo^c^ 2015 | Review | (27)	Policymakers, stakeholders	Government-recognized or public-funded national telehealth programs in Asia were searched along with their corresponding state of governance and management	Asian countries with funded telehealth programs were Bangladesh, India, Indonesia, Malaysia, Maldives, Philippines, and Sri Lanka	O: Challenges with governance, management, and sustainability of operations; lack of ICT governance framework
Mojica^a^ 2014 | Pilot | (28)	40 lower limb amputees in an urban community	The *Amputee Screening through Cellphone Networking* (ASCENT) application was designed to detect amputees in the community	ASCENT showed excellent overall agreement and inter-observer reliability among medical interns and health workers in the community. It was an easy and fast way to screen and refer amputees through internet-enabled asynchronous telerehabilitation	H: Need to train end-users O: Need to partner with a software developer T: Web-based app was Java-enabled, which could be slow and memory-consuming; non-editable referral once sent; relied on network signal
Sahu^a^ 2014 | Review | (29)	Patients with chronic diseases in Asia and Africa	Delivery of health information through cellphone	Mobile text messaging between patient and healthcare provider was convenient and effective in health monitoring, self-management of chronic diseases, delivery of individualized pharmaceutical care, medication adherence, and public health awareness	H: Need to adjust mindset of end-users, empower patients with medical knowledge in everyday language, and ensure them of confidentiality O: Limited evidence, especially on cost–benefit ratio, long-term benefits, and different study settings; lack of guidelines to ensure quality electronic delivery of healthcare; lack of standardized approach for the design, development, and evaluation of m-health technologies T: Undependable broadband internet speed; m-health technologies lack good display and adequate security controls; lack of intelligent algorithms to identify clinically significant events before notifying caregivers
Macrohon^a^ 2013 | Cohort | (30)	8 rural municipal health officers and 39 patients	Teleconsultation with a remote specialist using combined web- (Moodle™) and short messaging system (SMS)-based techniques	Referral via SMS was more common. High satisfaction was noted	H: Apprehensions on convenience, costs, sustainability, and privacy; unavailability and apprehensions of urban-based specialists in the specific field of expertise O: Low utilization of teleconsultation program; time-consuming process; little time to tele-refer amidst other clinical/administrative responsibilities; lack of community-based technical support; vague legalities and reimbursements of teleconsultations T: Variable internet bandwidth, network signal, and electricity across different rural areas; effort-requiring computer-based programs
Caranguian^c^ 2012 | Feasibility | (31)	Problem: The ISO/IEEE 11073 Personal Health Device Standards have not adequately addressed security and authentication of medical devices.	To address security, two approaches were tested: direct software implementation and use of embedded security modules (ESM)	ESM offered greater security advantage, such as secure keys storage	T: Lack of telemedicine system integrity
Fernandez-Marcelo^c^ 2012 | Mixed methods | (32)	Policymakers, researchers, educators, healthcare providers, telehealth enthusiasts	Review, key informant interviews, and conferences were done to explore e-health capacities in research, education, and service	Awareness of e-health was promoted by stakeholders	O: Lack of policies and standards, capability-building, and multi-sectoral collaborations
Macrohon^a^ 2011 | Feasibility | (33)	Rural patients	Teleconsultation program, consisting of cellphone- and web-based methods, used by rural physicians to refer cases to urban specialists	Majority used cellphone-based methods (texting more than calling)	H: Concerns about costs and waiting time to receive responses to referrals T: Need to boot-up equipment when using computers; variable broadband internet speed
Marcelo^c^ 2011 | Mixed methods | (34)	Problems existed in building internal capacity for telehealth in developing countries	Literature review and key informant interviews were done to explore partnerships, standards, and interoperability as components of health informatics programs	Developing countries needed to enhance capacities for m-health technologies	O: Difficult networking across archipelago; limited investments in building capacity for health informatics; government's slow adoption of health informatics standards and lack of collaboration with developed institutions due to social, political, and economic challenges; lack of human resources and training to support health informatics; lack of privacy frameworks and standards for interoperability T: Impractical conventional hardware (servers, workstations) in underserved areas with power fluctuations; inadequate technology infrastructure; licensed proprietary software limiting ability of local programmers to observe and improve software engineering practices
Marcelo^c^ 2010 | Review | (35)	Problem: Many health information systems (HIS) in developing countries fail during implementation	Existing international HIS were reviewed	Successful HIS and frameworks could serve as models for resource-constrained healthcare settings	O: Lack of or unsustainable HIS; non-adoption of existing successful frameworks
Alis^a^ 2009 | Feasibility | (36)	30 patients with pre-diagnosed cardiac pathologies	Real-time information on cardiac status of patients from a telemonitoring device was sent to off-site specialists via mobile phone	Patients were classified as follows: - normal (86.7% accuracy); - congestive heart failure (86.7%); - atrial fibrillation (80.0%)	T: Unreliable wired infrastructure; limited internet bandwidth for media transfer
Gavino^a^ 2008 | Case series | (37)	34 doctors-to-the-barrios	Doctors in rural areas referred patients to urban specialists through text	Extensive wireless network coverage (>90% of the country) in rural areas made mobile phones more accessible than web-based solutions	T: Inaccessible internet-based methods; limited (160) characters in a single text message using non-smartphones
Nguyen^c^ 2008 | Review | (38)	Policymakers, healthcare providers, telehealth enthusiasts	Review of healthcare informatics in Singapore, Cambodia, Malaysia, Thailand, Laos, Philippines, and Vietnam	Healthcare management varied widely in Southeast Asia from being well-developed in Singapore to being underdeveloped in Laos, affected by various political, economic, societal, cultural, and educational factors	O: Poor healthcare financing; lack of harmonization between private and public sectors; unsustainable e-health programs; lack of ICT support

a *Engaged patients and physicians in teleconsultation, teletherapy, telementoring, or telemonitoring*.

b *Focused on awareness and other factors affecting telemedicine or telerehabilitation*.

c *Related to telehealth-related governance, national policies, legalities, and ethics. ICT, Information and communications technology; ISO/IEEE, International Organization for Standardization/Institute of Electrical and Electronics Engineers*.

Teleconsultation meant that a patient consulted with a remote physician (16, 29). Teletherapy meant that a patient received instructions and home exercises demonstrated or supervised by a remote therapist (19, 20). There was one local study that involved physiatrists, physical therapists, occupational therapists, psychologists, and rehabilitation nurses in multi-disciplinary telerehabilitation sessions with a remote community (20). Another study involved speech-language pathologists (19), while two studies involved psychologists (17, 26). Telementoring meant that a remote specialist gave expert advice to a rural physician or healthcare worker co-located with a patient (20, 30, 33, 37). Telemonitoring meant that a gadget or web-based application facilitated asynchronous remote transmission of health-related information or patient reminders (17, 18, 28, 36). The most common electronic methods of conducting telerehabilitation (i.e., teleconsultation, teletherapy, telementoring, or telemonitoring) used in the local studies were mobile text messaging or short messaging system (SMS) (29, 30, 33, 37), followed by videocall and instant messaging through available social media platforms, such as Viber™ (16), Skype™ (20), or FaceTime™ (19). Two studies conducted teleconsultations through combined web- (i.e., Moodle™) and SMS-based services (30, 33). In general, positive experiences were noted from patients and rural physicians. The concerns raised, however, were mostly related to internet speed and data privacy issues.

The rest of the studies were related to telerehabilitation acceptance (Laron et al., unpublished; Mendoza et al., unpublished), telehealth-related governance (22, 32, 34, 35, 38), national programs or policies (23, 24, 27), legal issues (24), data privacy and security concerns (24, 31, 39) and ethical dilemmas (24, 25). Majority of the authors of these papers were affiliated with the National Telehealth Center of the National Institutes of Health at the University of the Philippines Manila. It was found that no Philippine law specific to telehealth has been approved yet according to Patdu and Tenorio (24). Nonetheless, there were initial efforts to lobby for telehealth by addressing funding, legal, ethical, and administrative challenges (23, 24, 32, 40).

### Challenges to Telerehabilitation

While Table 1 contains the human, organizational, and technical challenges cited in each study, Table 2 groups together similar challenges and organizes them into these three categories in order of frequency. In terms of human factors, the most commonly discussed challenges in the included studies were lack of acceptance of telehealth among stakeholders (in 9 studies), lack of knowledge and skills needed in e-health (6), and apprehensions related to data privacy (4). Among the organizational factors, which account for the highest percentage (42%) of the total frequency of citations of identified barriers, the most pressing were the lack of national e-health policies or laws (in 9 studies), health information systems framework (8), governance (5), and data privacy measures (5). Among all individual factors across categories, the internet was the overall number 1 challenge to telehealth in the Philippines, as mentioned in at least 10 studies.

**Table 2:** Frequency of human, organizational, and technical challenges to telerehabilitation in the Philippines cited in included studies.

**Challenges**	**References**	**Frequency**
**A. HUMAN FACTORS 41**		
1. Skepticism/lack of acceptance/resistance to change/negative attitudes	[(16–18, 21, 22, 29, 30); Laron et al., unpublished; Mendoza et al., unpublished]	9
2. Lack of technical or digital knowledge and skills; need for training	[(16, 17, 19, 20, 28); Mendoza et al., unpublished]	6
3. Concerned about data privacy/confidentiality/security	(17, 18, 29, 30)	4
4. Lack of awareness of telemedicine/telerehabilitation	(16, 17, 29)	3
5. Concerned about costs	(30, 33)	2
6. Concerned about national laws/legalities	(17, 18)	2
7. Inadequate rapport	(Laron et al., unpublished; Mendoza et al., unpublished)	2
8. Lack of patient participation/poor adherence	(22, 26)	2
9. Perceived inconvenience/time-consuming	(30, 33)	2
10. Concerned about appropriateness	Laron et al., unpublished	1
11. Concerned about effectiveness	Laron et al., unpublished	1
12. Concerned about efficiency	Laron et al., unpublished	1
13. Concerned about informed consent	(18)	1
14. Concerned about safety	Laron et al., unpublished	1
15. Concerned about sustainability	(30)	1
16. Lack of satisfaction	Mendoza et al., unpublished	1
17. Paper culture	(21)	1
18. Poor telecommunication skills	(22)	1
**B. ORGANIZATIONAL FACTORS 60**		
1. Lack of national e-health policies/laws/regulations	(22–25, 27, 29, 30, 32, 34)	9
2. Need for ICT infrastructure/HIS framework/partnerships	(23, 27–29, 32, 34, 35, 38)	8
3. Lack of governance/support	[(27, 32, 34); Laron et al., unpublished; Mendoza et al., unpublished]	5
4. Lack of platforms/measures that ensure privacy and security	(16, 23, 24, 30, 34)	5
5. Financing and reimbursement problems	(23, 30, 34, 38)	4
6. Lack of e-health resources	[(22, 30); Laron et al., unpublished; Mendoza et al., unpublished]	4
7. Lack of technical support	(20, 30, 34, 38)	4
8. Lack of telerehabilitation guidelines/standards	[(16, 19, 22); Mendoza et al., unpublished]	4
9. Lack of training for providers	[(23, 34); Laron et al., unpublished; Mendoza et al., unpublished]	4
10. Unclear accountability/roles/liabilities	(23, 25, 30)	3
11. Difficult implementation; unsustainable program; low utilization	(21, 30)	2
12. Lack of studies/evidence	[(29); Mendoza et al., unpublished]	2
13. Time-consuming process; busy work schedule	(20, 30)	2
14. Environmental constraints to telehealth	Mendoza et al., unpublished	1
15. Lack of exercise equipment	(20)	1
16. Lack of updated community-based rehabilitation policies	(19)	1
17. Lack of validated data collection tools/performance measures	Mendoza et al., unpublished	1
**C. TECHNICAL FACTORS 42**		
1. Slow internet/limited internet coverage	[(16, 19, 20, 29, 30, 33, 34, 36); Laron et al., unpublished; Mendoza et al., unpublished]	10
2. Difficult or time-consuming to use/sustain	(21, 30, 33)	3
3. Lack of security	(20, 29, 31)	3
4. Lacks interoperability	[(22); Laron et al., unpublished; Mendoza et al., unpublished]	3
5. Software limitations/inadequacies	(26, 28, 37)	3
6. Dependence on electricity	(30, 34)	2
7. Dependence on internet	(28, 37)	2
8. Difficult examination/treatment	(Laron et al., unpublished; Mendoza et al., unpublished)	2
9. Hardware failure/defects/limitations	[(34); Mendoza et al., unpublished]	2
10. Inadequate infrastructure	(34, 36)	2
11. Limitations of artificial intelligence	(18, 29)	2
12. Unclear video/display	(20, 29)	2
13. Expensive	(19)	1
14. Lacks capacity for empathy	(18)	1
15. Lacks contextualization	(22)	1
16. Lacks correlation with face-to-face assessment/treatment	(26)	1
17. Licensed proprietary software	(34)	1
18. Limited network coverage	(30)	1

*ICT, information and communications technology; HIS, health information systems*.

Since human factors pertain to internal challenges (or within the person) (41), majority of those listed in Table 2A are interrelated with one another and may contribute to skepticism. Several studies have evaluated or attempted to address the lack of awareness and acceptance of telemedicine among stakeholders. Research fora, stakeholders' meetings, campaigns, and conferences conducted by Fernandez-Marcelo et al. in 2012 stimulated awareness of telehealth in a wider scale locally (32). The National Telehealth Service Program of the Department of Health was an important milestone in spreading telehealth awareness in rural areas, as shown by Macrohon and Cristobal (30, 33) and Gavino et al. (37). Local studies by Leochico and Mojica (20), Leochico and Valera (16), and Mojica et al. (28) in the Philippine General Hospital sprung awareness of telerehabilitation in particular. Two unpublished reviews found positive attitudes and limited experience with telerehabilitation among allied rehabilitation professionals in developing countries (Laron et al., unpublished). However, no published study related to telerehabilitation knowledge, attitudes, and perceptions among healthcare professionals was found from the Philippines. The study by Mandirola-Brieux et al. stated that cultural factors played a role in the acceptance of e-health programs (21). A systematic review on the role of telehealth in African and Asian countries showed that mobile text messaging was the most commonly accepted telehealth method among patients with chronic diseases (29).

There were several factors that were classified into more than one category, depending on the context in which the factor was discussed in individual studies. For instance, challenges related to national laws and guidelines, albeit more commonly discussed as an organizational factor, were contributory to human factors (i.e., apprehensions and various concerns). Meanwhile, issues on data privacy or security were listed under each category. Another recurring theme across all categories was related to technical aspect of e-health, cited as lack of digital knowledge and skills (under human factors), lack of technical support and training (under organizational factors), and technologies that were difficult to use, along with software and hardware issues (under technical factors).

## Discussion

Our study found 53 unique, albeit interrelated, challenges in the literature that could affect the emergence of telerehabilitation in the Philippines. This review was driven by the difficulties experienced first-hand by the authors during the pre-implementation and implementation periods of telerehabilitation in local private and public healthcare settings in response to COVID-19. Probably similar to most developing countries without pre-existing telerehabilitation guidelines, rehabilitation providers in the Philippines were generally unprepared and apprehensive to adopt telerehabilitation in their practice. Evidences in this review helped us name the felt barriers to telerehabilitation and telehealth in general and categorized them into human, organizational, and technical factors in order of frequency. Overall, organizational factors accounted for the highest number of citations similar to a previous systematic review (42), while the most commonly cited specific factor across all categories was internet connection, as experienced in low- or middle-income countries (43).

Telerehabilitation literature in the Philippines is limited to feasibility studies and case reports. Despite scarce local evidence and experience, telerehab was deemed feasible even before the pandemic to perform remote teleconsultation, teletherapy, telementoring, or telemonitoring mostly for indigent patients in rural areas. As of writing, however, no local telerehabilitation document exists to operationally define various interchangeable terms, such as telehealth, telemedicine, telerehabilitation, teletherapy, telepractice, and telecare among many others used in the different rehabilitation disciplines. More so, there is no guideline on telerehabilitation principles, scope of services, procedure, and regulations that can be applicable across various rehabilitation professional organizations in the country.

Several success stories of national telerehabilitation programs abroad can inspire the eventual emergence of telerehabilitation in the Philippines. For instance, Canada and Australia use telerehabilitation to enhance access across vast geographical landscapes and minimize economic barriers by reducing travel time and costs (44, 45). Meanwhile, India, a lower-middle-income country (6), has a teleneurorehabilitation program to remotely provide cost-effective services amidst limited medical resources (46). Each country that has adopted telerehabilitation even before the pandemic acts according to the needs of its people and healthcare system.

The rehabilitation needs of the growing population from all over the Philippine archipelago cannot always be addressed face to face because of the barriers of distance, time, costs, manpower, and resources. Center-based rehabilitation services are limited, with more than 50% of the facilities located in urban areas of the National Capital Region (NCR) (47). There are only 216 fellows of good standing recognized by the Philippine Academy of Rehabilitation Medicine, 78 of whom have their primary practice in NCR (Figure 1) (47). Among physical therapists (PTs), there are 5,327 members of the Philippine Physical Therapy Association out of the 14,610 licensed PTs (48). Meanwhile, there are 2,985 occupational therapists, 673 speech-language pathologists, and 53 prosthetists–orthotists in the country (49, 50). Included in these numbers are those who might have migrated abroad, changed career, or retired. The greatest proportion of rehabilitation workforce remaining in the Philippines is based in Luzon (51).

Various local rehabilitation services, such as community-based programs, have been in place to support PWD throughout the country. However, efforts to empower rural communities and PWD have been hampered by several challenges, such as low accessibility, high costs, low utilization, and low sustainability (52). Amidst social distancing due to COVID-19, face-to-face rehabilitation might not adequately and safely cope with the continuing demand of PWD. A potentially viable solution is telerehabilitation, but it is also not without challenges.

In line with the Philippine e-Health Systems and Services Act, feasibility and cost-effectiveness studies should be done prior to implementation of telehealth-related programs in healthcare facilities (53). In addition, awareness campaigns, workforce training, capacity-building, and policy-updating are also important measures to ensure sustainable programs (23, 32). In relation to local telerehabilitation experience, however, these crucial steps were bypassed during the COVID-19 pandemic to urgently come up with interim guidelines.

When planning a telerehabilitation program, it should be emphasized that guidelines vary from one healthcare setting to another, depending on human, organizational, and technical factors. First, human (internal) factors include telerehabilitation awareness, acceptance, readiness, knowledge, and skills [Laron et al., unpublished; (41)]. Local studies on these interrelated human factors among different stakeholders (i.e., patient, family or caregiver, healthcare provider, policymakers, third-party payers) are recommended. Second, to address organizational (external) factors, the following are recommended: lobbying for administrative support and funding, formulation of best practice guidelines, work reorganization, agreement on payment schemes and reimbursements, and measures to protect data privacy and safety of stakeholders (23, 27, 53). Lastly, technical factors should be addressed by improving the quantity and quality of tangible (i.e., telerehabilitation equipment and technical support) and intangible e-health resources (i.e., technical skills, information and communications framework or “infostructure”) (22). Understanding and addressing such factors are key to successful telerehabilitation initiatives.

As evident during the pandemic, videoconferencing has become relatively more feasible locally compared to earlier years. During the first quarter of 2017, Akamai, a recognized cloud data network monitoring internet traffic, reported that the Philippines had the largest quarterly increase in internet speed at 26% in the Asia-Pacific region (54). Still, however, the country had the lowest average connection speed at 5.5 megabits per second (Mbps), compared to the global speed of 7.2 Mbps (54). In addition to its slow speed, the internet in the Philippines has not always been cheap with mobile cellular and fixed broadband services amounting to 22.24 and 51.59 USD per month, respectively (55). Although the country has been working on national reforms toward universal internet access (56), we have yet to see improvements in the technology to facilitate e-health access.

As strengths of this study, we were able to contribute to the limited knowledge of telerehabilitation facilitators and barriers in a developing country. We structured our paper following the PRISMA guidelines. We attempted to increase the number of included studies by handsearching of gray literature. We analyzed 25 studies and extracted the human, organizational, and technical challenges that might be applicable not only in the Philippines but also in other resource-limited countries. In terms of limitations, a more thorough handsearching of gray literature from other institutions across the archipelago and inclusion of studies from other developing countries could have been done. A more objective screening process could have also increased the number of analyzed studies. Although we attempted to control for this limitation by having more than one reviewer screening each study, potential bias toward rehabilitation medicine has excluded studies from other specialties, whose experiences could have also been rich data sources on telehealth challenges. Another factor that might have influenced our results was individual judgment in analyzing the studies and extracting the barriers and categorizing them into human, organizational, and technical factors. Nonetheless, we tried to address this limitation by consensus meetings. Lastly, the challenges cited in this paper were solely based on secondary data; hence, future large-scale descriptive and analytical studies gathering primary data are recommended.

As more stakeholders recognize the value of telerehabilitation, catalyzed by the COVID-19 pandemic, more efforts can be made to address the various challenges besetting the emergence of telerehabilitation in the country. Researches on telerehabilitation and policy changes through Delphi method can help us respond better to the World Health Organization's *Rehabilitation 2030 Call to Action* to improve access to rehabilitation services (10, 42, 57). A lot of work has yet to be done to address the human, organizational, and technical challenges to telerehabilitation, but we can be guided by existing local and international evidences, along with experts in telehealth and medical informatics, to avoid costly and time-consuming trial-and-error attempts.

## Data Availability Statement

The original contributions presented in the study are included in the article/supplementary material, further inquiries can be directed to the corresponding author/s.

## Author Contributions

CL conceived the idea and wrote the initial drafts and final revisions of the manuscript. AE, SI, and JM made substantial contributions in the content and format of the revised versions. All authors contributed to the article and approved the submitted version.

## Conflict of Interest

The authors declare that the research was conducted in the absence of any commercial or financial relationships that could be construed as a potential conflict of interest.
